# A scaffolding intervention to improve self-efficacy in source-based argumentative writing

**DOI:** 10.3389/fpsyg.2024.1454104

**Published:** 2024-11-08

**Authors:** Besma Allagui

**Affiliations:** Rabdan Academy, Abu Dhabi, United Arab Emirates

**Keywords:** argumentation, intervention, scaffolding, self-efficacy, source-based writing

## Abstract

This study examined the role of scaffolding as temporary support offered by an educator to help students complete a learning task that would be challenging to accomplish without this support. Although there is a great deal of research in (L2) second language writing on the effect of scaffolding on improving students’ writing outcome variables (e.g., organization, coherence, content, and overall writing performance), few studies have explored the contributions of scaffolding to learners’ emotions and psychological variables. Using a double pre-test post-test design, this quasi-experimental study investigated the effect of a scaffolded writing intervention on students’ self-efficacy beliefs (i.e., confidence in their ability) in source-based argumentative writing. We analyzed the students’ (*N* = 50) source-self-efficacy beliefs 3 weeks before the intervention, immediately before the intervention, and immediately after the intervention. At the end of the intervention, students’ performance was measured using a scoring rubric containing key criteria in source-based argumentative writing. A post-study semi-structured interview was conducted with participating students (*N* = 13) to further understand their experience with the scaffolding intervention. The analyses demonstrated that the intervention had a positive and significant impact on students’ self-efficacy beliefs of their abilities to organize ideas, summarize different sources, and revise their essays. There were no significant changes in the participants’ beliefs about skills related to deciding if the evidence from different sources is strong, finding weaknesses in the arguments presented in different sources, and writing a counterargument. Furthermore, correlational analysis using post-test self-efficacy overall score and students’ writing performance scores on four major aspects (idea development, organization, source use, and language use) revealed a positive relationship between self-efficacy and two aspects of writing (source use and organization). Students’ interview results further confirmed the effectiveness of the intervention in enhancing the participants’ self-efficacy beliefs and performance in source-based argumentative writing. These findings highlight the importance of scaffolding strategies targeting self-efficacy to improve confidence in source-based writing and hence writing performance.

## 1 Introduction

Source-based writing is a “hybrid” type of writing in which learners integrate one or multiple sources to create a coherent new text (Spivey and King, 1989). Such writing facilitates knowledge transformation because it engages students in complex and iterative activities, including reading, rereading, selecting, organizing, and integrating information from multiple texts which are essential for effective communication (Leijten et al., 2019; Tarchi et al., 2023; Leijten et al., 2019; Tarchi et al., 2023). Moreover, source-based writing is often described as more authentic and more useful particularly for L2 learners than independent writing (i.e., prompt-based) because it provides students with source materials to draw from, offering background information and vocabulary, which helps them communicate more effectively (Weigle, 2004).

Despite these positive contributions, source-based writing is challenging even for advanced undergraduate university students (Allagui, 2023; Leijten et al., 2019; Plakans, 2008; Plakans and Gebril, 2012; Tarchi et al., 2023). Prior research in source-based writing revealed that L2 students struggle with synthesizing information from multiple texts, maintaining their own voice while incorporating source material (Cumming et al., 2016; Leijten et al., 2019), and distinguishing between paraphrasing, summarizing, and direct quotes (Wette, 2017; Wette, 2019; Wette, 2023; Zhou et al., 2022). These difficulties are compounded when students are writing in the argumentative genre, as they must not only present information but also construct and support a coherent argument and address counterarguments (Marttunen and Kiili, 2022; Nussbaum and Schraw, 2007; Wolfe and Britt, 2008). Thus, there appears to be a discrepancy between the potential benefits of source-based writing and students’ ability to demonstrate effective use of sources in their writing. This discrepancy highlights the need for instructional strategies that allow learners to practice source integration skills in a supportive environment.

One instructional strategy to help learners overcome source-based writing difficulties is *scaffolding*. Scaffolding consists in providing temporary support that gradually helps students develop their performance and autonomy (Belland, 2014; Reiser, 2018; Shvarts and Bakker, 2019; Wood et al., 1976). The effectiveness of scaffolding in source-based L2 writing has yet to be determined, as past studies have tended to focus on applications of various scaffolds in independent writing contexts (e.g., Britt and Aglinskas, 2002; Cui et al., 2021; Doolan and Fitzsimmons-Doolan, 2023; Lee and Evans, 2019; Luna et al., 2023; Mateos et al., 2008). These studies have provided evidence to show that students benefited from scaffolding strategies involving collaborative practice (e.g., Mateos et al., 2008), explicit instruction about organizing and integrating information from sources (e.g., Britt and Aglinskas, 2002), teacher feedback (e.g., Cui et al., 2021), peer feedback (e.g., Lee and Evans, 2019), and modeling the process of writing from sources (e.g., Doolan and Fitzsimmons-Doolan, 2023; Luna et al., 2023). Furthermore, these studies reported that students were generally satisfied with scaffolding strategies adopted by the teacher to support their writing. However, the results remain inconclusive, and there is still a debate on the fragmented nature of these scaffolding strategies, as researchers have developed their own framework.

Additionally, L2 writing researchers still need to disentangle the impact of scaffolding strategies on students’ self-efficacy beliefs when completing source-based writing assignments. Self-efficacy or individuals’ belief in their ability to accomplish a complex task is the main construct in the socio-cognitive theory of learning (Bandura, 1997; Zimmerman, 2002). Self-efficacy plays a pivotal role in learning and development as it influences individuals’ motivation, persistence, and overall task performance (Bandura, 1997). A plethora of studies in the realm of L2 writing confirmed that self-efficacy is a major predictor of writing performance across different genres (narrative, argumentative, etc.…) (Bruning and Kauffman, 2016; Klassen, 2002; Mitchell and McMillan, 2018; Pajares, 2003; Pajares et al., 2007; Raoofi et al., 2017). Therefore, given the known positive relationship between self-efficacy and writing performance as well as the extensive literature on the positive contribution of scaffolding on writing performance, it is important to investigate whether scaffolding can influence self-efficacy beliefs in source-based writing. This investigation is especially important because of dearth of research on the psychological contributions of scaffolding. While previous scaffolding interventions in L2 writing have occasionally addressed self-efficacy beliefs, they have not made it a primary focus for improvement (*see* Mitchell et al., 2023 *for an overview*). To the best of our knowledge, no study has specifically designed an intervention aimed at increasing self-efficacy in L2 source-based writing.

The present study aims to explore the impact of *an intentionally-designed scaffolding intervention* on increasing *students’ self-efficacy in source-based argumentative writing*. Our intervention involved breaking down the larger argumentative writing assignment into a sequence of smaller assignments and providing students with teacher feedback before and after each assignment to further guide their progress. Additionally, we aim to explore the relationship between *self-efficacy in source-based argumentative writing* and *source-based argumentative writing performance* to provide empirical evidence that can support the design and implementation of self-efficacy-based scaffolding. If a strong correlation is found, educators can confidently focus on self-efficacy-building interventions as a means to enhance writing. Furthermore, we aim to obtain students’ opinions on the effectiveness of the intervention in enhancing source-based argumentative writing performance and self-efficacy beliefs. This investigation is particularly important to further promote the role of scaffolding in source-based writing contexts. Conclusions drawn from the study could inform practitioners and educators of the potential of scaffolding for helping students write better source-based argumentative essays.

## 2 Review of the literature

This literature review will provide an overview of self-efficacy, scaffolding, and discuss various self-efficacy-based scaffolding interventions in L2 writing. It will then explore scaffolds designed to increase self-efficacy in source-based writing. Gaps and limitations in the extant literature will be further explained.

### 2.1 Self-efficacy

Bandura (1986) defined self-efficacy as “people’s judgments of their capabilities to organize and execute courses of action required to attain designated types of performances” (p. 391). This concept plays an important role in learning, effort, and persistence. As Pajares et al. (2000) noted, learning is “not just a matter of how capable you are, it’s also a matter of how capable you think you are” (p. 13). Learners with low self-efficacy are likely to avoid learning when faced with difficulties, and find it difficult to recover from failure (Schunk and DiBenedetto, 2022). In contrast, learners with high self-efficacy set ambitious goals, maintain a strong commitment to accomplishing them, and demonstrate perseverance in the face of obstacles (Zimmerman, 2002).

A comprehensive understanding of self-efficacy involves recognizing the four key sources from which individuals develop beliefs about their capabilities: enactive mastery, vicarious experience, social persuasion, and physiological and emotional state (Bandura, 1986). Enactive mastery develops from performing a particular task successfully and has been shown to play the most influential role in determining self-efficacy (Pajares et al., 2007). Vicarious experiences are derived from observing others’ successes thereby enhancing their own belief in their abilities. Verbal persuasion refers to encouragement and constructive feedback from mentors or teachers which can bolster an individual’s confidence. Finally, an individual’s physiological and emotional states—such as feelings of stress or fatigue—also play a significant role in influencing self-efficacy.

Numerous empirical studies have established a positive relationship between self-efficacy and L2 writing performance, indicating that higher self-efficacy can lead to improved writing outcomes (Jalaluddin, 2013; Klassen, 2002; Kormos, 2012; Mitchell and McMillan, 2018; Xu et al., 2023). A meta-analysis by Usher and Pajares (2008) revealed that enactive mastery was the most significant predictor of writing self-efficacy, while other influences such as vicarious experiences and verbal persuasion were found to be inconsistent predictors. Furthermore, prior L2 writing research found that self-efficacy is not a stable construct; it fluctuates based on task demands and complexity (Li et al., 2023; Mitchell and McMillan, 2018; Mitchell et al., 2023). This finding suggested that self-efficacy can be developed through targeted instruction and intentional feedback.

### 2.2 Scaffolding

The concept of scaffolding emerged from Vygotsky’s (1987) notion of zone of proximal development (ZPD) which views learning as a social act rather than an independent endeavor. Vygotsky (1987) argues that learning must be guided and supported by adult modeling and corrective feedback. While there are many definitions of scaffolding in education, the most widely accepted definition of scaffolding refers to a “process that enables a child or novice to solve a problem, carry out a task or achieve a goal which would be beyond his unassisted efforts” (Wood et al., 1976, p. 90). In this process, the teacher acts as a facilitator who offers temporary support and gradually fades away. Van de Pol et al. (2010) further elucidates the process of scaffolding through three key steps: contingency, intersectionality, and transfer of responsibility. Contingency involves continuously assessing students’ abilities; intersubjectivity refers to a shared understanding or common framework developed among learners during collaborative problem-solving; and transfer of responsibility which emphasizes that learners should gradually take ownership of their learning process.

There are several forms of scaffolding strategies but Saye and Brush (2002) classified them into hard and soft scaffolds. Hard scaffolds refer to “supports that can be anticipated and planned in advance” (e.g., graphic organizers, rubrics, multimedia resources) and soft scaffolds refer to “situation-specific assistance offered by a teacher or peer to facilitate the learning process” (e.g., probing questions, just-in time support) (Saye and Brush, 2002, p.81). The forms of scaffolding were broadened to include support that is presented at the macro, meso and micro levels of the curriculum (Boblett, 2012). The macro level encompasses the sequencing of modules; the meso-level incorporates the purposeful sequencing of class activities and assignments; and the micro level includes just-in-time support. Understanding these distinctions is crucial for effectively designing scaffolding interventions that meet diverse learner needs.

Moreover, it is essential to determine the functions of various scaffolding strategies (Azevedo and Hadwin, 2005; Mahan, 2022). One main function is cognitive scaffolding which provides hints, support, and assistance necessary for problem-solving and learning. Another function is metacognitive scaffolding which focuses on helping learners plan, monitor, and regulate their own learning processes. A third function is motivational scaffolding which aims to enhance students’ motivation and reduce negative emotions when completing a complex task. Educators should pay greater attention to the specific cognitive, metacognitive, and motivational functions of scaffolding strategies in order to create tailored support that directly addresses the diverse needs of their students.

### 2.3 Previous studies

Numerous studies across various disciplines have examined the role of scaffolding interventions specifically designed to enhance self-efficacy beliefs (Cui et al., 2021; Dominguez and Svihla, 2023; Ha et al., 2021; Huang et al., 2020; Koskinen et al., 2023; Li et al., 2023; Mandouit and Hattie, 2023; Zhou et al., 2022). Over the years, there has been an increasing interest in examining the effect of scaffolding on L2 writing self-efficacy (Campbell and Batista, 2023; Chen et al., 2021; Chung et al., 2021; Cui et al., 2021; Duijnhouwer et al., 2010; Falardeau et al., 2024; Graham et al., 2005; Huang et al., 2020; Lee and Evans, 2019; Panadero et al., 2023; Teng, 2022; Vandermeulen et al., 2023). Overall, the extant literature shows that four primary strategies have significant potential in supporting students’ writing self-efficacy: explicit instruction, teacher feedback, peer feedback, and rubrics and exemplars:

### 2.4 Scaffolding in the form of explicit instruction

Explicit instruction involves teacher guidance and explicit attention to elements of the writing process such as goal setting, drafting and revising (Chen et al., 2021; Graham et al., 2005; Graham and Perin, 2007). A meta-analysis conducted by Graham and Harris (2017) suggested that explicit instruction has a significant impact on students’ self-efficacy in writing by providing strategies that foster confidence and autonomy in the writing process. Chung et al. (2021) conducted a year-long writing intervention focused on revision in a Chinese context. Their findings emphasized the importance of self-assessment, planning, goal-setting, and reflective practices, with participants in the treatment group showing significant gains in self-efficacy and post-test writing performance. Similarly, Teng (2022) found that integrating self-regulated learning strategies with formative assessment improved writing quality and motivational beliefs, including self-efficacy. Additionally, research suggested that explicit instruction is even more effective when assisted by peers (Falardeau et al., 2024; Mohammadi et al., 2023). For example, Falardeau et al. (2024) investigated the impact of explicit instruction with and without peer feedback over a sequence of lessons focused on argumentative writing, feedback, the structure of opinion papers, and understanding of reader characteristics. Results indicated that the experimental group showed increased self-efficacy, suggesting that engaging in social activities like pair or group work further provided emotional support to students.

### 2.5 Scaffolding in the form of teacher feedback

Feedback is a form of social persuasion that plays a crucial role in shaping students’ self-efficacy beliefs (Bandura, 1997). There is much evidence in the field of L2 writing that teacher feedback promotes self-efficacy (Cui et al., 2021; Hyland and Hyland, 2001, 2019; Li and Zhang, 2022; Lundin et al., 2023; Mitchell and Pessoa, 2017; Razmi and Ghane, 2024; Ruegg, 2018). In a study of the impact of writing center consultations on students’ self-efficacy, Lundin et al. (2023) demonstrated that teacher feedback enhances students’ self-efficacy through four empathy-based approaches: listening, translating, advising, and motivating. However, research on teacher feedback also revealed that not all types of feedback can be beneficial (Banaruee et al., 2017; Banaruee et al., 2018). Banaruee et al. (2017) investigated the impact of receiving either explicit or implicit feedback during a 15-session writing course. Pre-tests and post-tests were conducted to evaluate improvements in writing proficiency. Results demonstrated that extroverts benefited more from explicit feedback suggesting that feedback should be contingent (i.e., tailored to the specific needs and personality traits of learners). The study conducted by Duijnhouwer et al. (2010) provides further empirical support for the importance of contingent feedback. Their research investigated how providing learners with feedback improvement techniques and reflective practice impacts self-efficacy among university students. Findings demonstrated that students in the experimental group significantly increased their self-efficacy beliefs more than the students in the control group.

### 2.6 Scaffolding in the form of peer-feedback

Peer feedback can be integrated into the classroom to scaffold L2 writing instruction. Various studies demonstrated that peer feedback can provide learners with vicarious experience which is one of the primary sources of self-efficacy (Campbell and Batista, 2023; Lee and Evans, 2019; Rodríguez-González and Castañeda, 2018 Yu and Hu, 2017). In the Chinese context, Lee and Evans (2019) investigated the impact of peer feedback on second language (L2) writing self-efficacy among 110 Chinese undergraduate learners of English. Participants were divided into treatment groups—face-to-face and computer-mediated discussions—and a comparison group, with data collected through questionnaires and interviews to assess changes in self-efficacy and the perceived usefulness of feedback. The results indicated that giving (but not receiving) peer feedback enhanced writing self-efficacy directly. Providing feedback enabled learners to observe their peers’ successful performance and make inferences about their own abilities which resulted in positive self-efficacy beliefs.

### 2.7 Scaffolding in the form of rubrics and exemplars

Using exemplars and rubrics is also considered a source of vicarious experience (Lipnevich et al., 2023; Schunk and DiBenedetto, 2022). In a review of 23 studies examining the effect of rubric use on self-efficacy beliefs, Panadero et al. (2023) suggested that providing students with examples of success offers several cognitive and motivational benefits. In an L2 writing setting, Andrade et al. (2009) examined the effect of using rubric on primary students’ (boys and girls) self-efficacy. Results revealed that using rubrics can help students evaluate their own capability to manage the task. Girls’ self-efficacy, however, was higher than boys’ self-efficacy and long-term rubric use associated only with the self-efficacy of girls.

To the best of our knowledge, few studies have examined the impact of various scaffolds intentionally designed to enhance self-efficacy in L2 source-based writing. Vandermeulen et al. (2023), for example, reported the positive impact of providing students with exemplars and teacher feedback. The results demonstrated that students displayed greater investment in writing behaviors. However, the study did not specifically measure self-efficacy beliefs. In an effort to understand the impact of scaffolding on self-efficacy beliefs in source-based writing, Zhou et al. (2022) conducted an intervention over a 10-week course using a sequence of regulatory activities. These activities involved analyzing source texts from textbooks as models to guide students through selecting, organizing, and connecting ideas, along with summarizing and paraphrasing skills. Additionally, participants’ written texts were used as models to identify and address common challenges in integrating sources into their writing. Their pre-test/post-test results demonstrated that this type of instructional scaffolding facilitated mastery as students showed increased confidence in applying the knowledge and strategies taught. However, the intervention did not specifically target self-efficacy. Van Blankenstein et al. (2019) developed an intervention specifically aimed at enhancing writing self-efficacy in source-based writing. The study measured students’ self-efficacy at three points during an undergraduate research project, finding significant increases throughout the year. The largest gains occurred after students completed the introduction to their research paper. The authors suggested that mastering a challenging task like writing an introduction (enacted mastery), combined with teacher feedback (social persuasion) and reading research articles as models (vicarious experience), and significantly boosted students’ self-efficacy.

### 2.8 Summary

To sum up, the literature on the effect of scaffolding on enhancing L2 writing self-efficacy appears to be disparate as researchers adopted different strategies and created their own frameworks. Additionally, although preliminary evidence supports the value of self-efficacy-based interventions in several domains including L2 writing, there has been little focus on the design of scaffolding interventions targeting students’ source-based L2 writing self-efficacy. Moreover, while some studies demonstrated the positive effect of social persuasion and enacted mastery on source-based writing self-efficacy, there is lack of understanding of the combined effect on students’ self-efficacy beliefs from four sources: mastery experience, vicarious experience, social persuasion, and physiological and emotional states. Researching the combined effect of the four sources of self-efficacy might be a promising avenue to enhance self-efficacy beliefs of the complex task of source-based writing.

This dearth of knowledge on the effectiveness of scaffolding on L2 source-based writing self-efficacy in addition to a lack of understanding of the combined effect of four sources of self-efficacy provided the motivation for the present study.

## 3 Methodology

### 3.1 Design

This study used a mixed-method approach to extend the previous research on the role of scaffolding through a sequence of written assignments associated with teacher feedback in enhancing self-efficacy and performance in source-based argumentative writing. Additionally, we aimed to obtain students’ opinion on the effectiveness of the scaffolding strategy in enhancing source-based argumentative writing performance and self-efficacy beliefs.

To measure the impact of the scaffolding strategy on students’ self-efficacy beliefs, the self-efficacy survey was designed using items related to source-based argumentative writing. The survey was administered three times during the semester: two weeks before the start of the intervention, immediately before the intervention and immediately after the intervention. The main advantage of a double pre-test design is that it helps provide evidence that can be used to refute the phenomenon of regression to the mean and confounding variables as alternative explanations for any observed association between the scaffolding intervention and the post-test results. If both pre-test 1 and pre-test 2 are similar, then, any significant changes from pre-test 2 to the post-test should be due mainly to treatment effects. Compared to randomized pre-test/post-test design, the double pre-test design is very strong in internal validity (Little et al., 2020). It is not susceptible to selection and maturation effect that can threaten the validity of randomized pre-post-test experiments due to differences that may exist between the two groups before the intervention.

To measure the role of the scaffolding strategy in students’ writing performance, we relied on an examination of the relationship between students’ writing performance measured at the end of the intervention and post-test self-efficacy overall score. Instead of measuring students’ writing performance in pre-test and post-test occasions, this approach can make more accurate predictions about the role of the scaffolding strategy in enhancing writing performance by examining how confident students feel in their abilities post-intervention and how this increased self-efficacy correlates with improvements in their actual writing performance.

To gain insights into students’ perspectives on the scaffolding strategy, post-study semi-structured interviews were conducted with a sample of students (*N* = 13). These interviews provided qualitative data on the perceived effectiveness of the scaffolding strategy in enhancing source-based writing performance and self-efficacy beliefs. The study attempted to answer the following research questions:

Question 1: To what extent does the use of a self-efficacy-based scaffolding strategy improve students’ self-efficacy beliefs?

Question 2: To what extent do the students’ self-efficacy beliefs relate to students’ source-based argumentative writing performance?

Question 3: How do the students perceive the effectiveness of using a self-efficacy-based scaffolding strategy in enhancing students’ source-based argumentative writing performance and self-efficacy beliefs?

### 3.2 The participants and context

The participants (*N* = 50) were Year 1–2 college students enrolled in programs in Homeland Security, Integrated Emergency Management, and Policing and Security programs in a public university in the UAE. They were selected using a convenience sampling technique from three intact academic writing classes. The course in which the students were enrolled was designed to give students practice into writing evidence-based argumentative essays, responding to readings and incorporating sources into their essays. The course took place once per week for two hours and 30 min. Each session emphasized that good writing requires the use of sources, the ability to incorporate sources, and the avoidance of plagiarism.

Participants were all male Emiratis aged 18–24 years coming from the seven Emirates. They spoke Arabic as their first language. They all graduated from local and international high schools in the UAE. The Arab Knowledge Report 2010–11 (United Nations Development Programme, 2012) revealed that 12th-grade students in schools across Abu Dhabi and Dubai scored an average of 5 out of 25 on a written communication assessment indicating a major weakness in writing skills. The PISA (2022) report (Organisation for Economic Co-operation and Development, 2023) found that only 5% of students in the UAE reached the top performance levels (Level 5 or higher) in reading. Both reports highlighted potential challenges that students may face in academic writing classes. However, to be admitted to higher education, students must achieve a minimum score of 5.5 at the IELTS (International English Language Testing System) exam which suggests that they possess the necessary skills to understand and communicate in English at an intermediate-upper intermediate level. It is worth mentioning that participation was voluntary and each student signed a consent form before the start of the study. The key characteristics of the 50 students participating in the study are summarized in Table 1.

**Table 1:** Key characteristics of the students participating in the study.

Gender	Males
Age	18–24
Year in college	1–2
Information literacy	All had good IT skills; each student took at least two classes in ICT before
English language proficiency	5.5 or more at IELTS

(IT) Information Technology; (ICT) Information and Communication Technology.

In addition, to the above indicators, students who consented to participate were assessed on their writing performance using their previous semester writing samples (summaries and opinion essays) to establish a baseline. Students’ samples were assessed a common 5-point rubric specifically designed for the study which provided a clear description of student’s writing in four areas: content, organization, language use and source use. This helped identify any existing differences between the students that could affect their writing performance. It also served as a diagnostic test of the main writing issues. To verify accuracy, a trained and independent rater reviewed the scores given by the researcher. The Cohen’s Kappa test (Field, 2009) revealed a high level of agreement between the raters (*κ* = 0.87), suggesting that the scoring was reliable. Generally, the low standard deviations suggested that the students had a homogeneous overall writing performance (summary: *M* = 11.23, *SD* = 0.13; Opinion essays: *M* = 9.04, S*D* = 0.24). By scrutinizing the written samples, it was revealed that problems the students faced mainly fell into five categories: (1) lack of clear organization, (2) patchwriting (i.e., many ideas are not well-paraphrased), (3) clarity (4) lack of evidence; and (5) lack of counterarguments and refutations.

### 3.3 Materials

#### 3.3.1 The sequence of written assignments

It was implemented in a 16-week academic writing course focused on enhancing students’ reading and writing skills. As part of the course, students were required to complete a source-based argumentative essay writing assignment.

To support student writers in their development of the final source-based argumentative essay, a series of assignments were designed. The purpose was to break the final essay assignment into shorter assignments focusing on pre-writing, evaluating sources, selecting evidence, summarizing, synthesizing evidence, outlining, and drafting. The series of assignments started with a proposal of an argumentative source-based essay, an annotated bibliography, and an outline culminating in a final source-based argumentative essay.

The proposal consisted of creating a 1-page argumentative essay that they will write at the end of the course. In this proposal, they should briefly explain why they chose a certain topic (the selection of topics was provided). They were required to state their position and list two ideas in favor of this argument. Also, they were asked to outline information (facts, statistics, and examples) that they would need to find to support their claim.

The second assignment consisted of a bibliography. They were required to create annotated bibliographies for three sources related to the topic for the argumentative essay using APA citation. They were provided with examples of strong and weak annotated bibliographies and targeted feedback. The third assignment consisted in a final draft. The students were required to write an outline of an argumentative essay that presented two arguments in favor of a particular issue and one counterargument against it. The final draft of the essay needed to include appropriately cited paraphrased information and a minimum of three APA references. Students should write a minimum of 750 words. Table 2 shows the sequence of assessments.

**Table 2:** The sequence of assignments.

Macro-level	Micro-level
Topic proposal	Choosing a research topic
	Identifying preliminary sources
	Examining models
	Pre-submission instructor feedback
	Post-submission instructor feedback
Annotated bibliography	Finding credible sources and evaluating sources
	Reading and note-taking, writing summaries
	Examining models
	Pre-submission instructor feedback
	Post-submission instructor feedback
Final argumentative essay	Outlining the final essay
	Examining models
	Drafting the introduction and thesis statement
	Writing body paragraphs using the outline as a guide
	Integrating evidence and citations correctly
	Drafting the conclusion to reinforce the argument
	Pre-submission instructor feedback
	Post-submission instructor feedback

This approach aligns with four sources of self-efficacy: vicarious experience, mastery experience, social persuasion, and emotional states. By breaking down the larger assignment into manageable steps students experience reduced anxiety and enhanced motivation (emotional states). By observing successful models (vicarious experience) students are able to estimate how they will perform. By completing each assignment successfully, students move on to the next assignment with beliefs in their prior success (mastery experience). Additionally, by receiving continuous and constructive teacher feedback (social persuasion) students reinforce their belief in their abilities.

#### 3.3.2 Feedback types

Students submitted all assignments through Turnitin to minimize plagiarism. Feedback was provided before submission and after submission. Before submission of each assignment, students received feedback from their teacher and peers on the content and structure of their writing. After submission, the teacher provided more targeted feedback using three methods: (1) the quick marks on Moodle which allowed the teacher to use pre-set feedback to address common issues on each assignment, (2) a scoring multiple-trait rubric which showed students exactly how their work was evaluated and where they met or fell short of the expected standards, and (3) a summative comment which offered students a holistic view of their performance, highlighting major strengths. The criteria used in each rubric are outlined in Table 3.

**Table 3:** Rubric criteria.

Macro-scaffolding steps	Rubric criteria
Topic proposal	clarity and thoroughness of the topic chosen the strength and support of the position statement the use of standard writing conventions the ability to identify all essential information for the essay
Annotated bibliography	accurate and clear summaries of sources a clear explanation of their relevance and usefulness logical and coherent organization with correct cohesive devices minimal grammatical errors correct APA citation formatting
Final essay	The clarity and engagement of the introduction, the restatement and summary in the conclusion the support and explanation of arguments and counterarguments with relevant evidence the organization and use of cohesive devices, minimal grammatical errors correct APA formatting of in-text citations and sources

#### 3.3.3 Source-based writing self-efficacy survey

In his guidelines for developing self-efficacy scales, Bandura (1997) explains that self-efficacy is domain-specific and should be assessed using the specific skills relevant to the particular domain rather than on unrelated skills or general skills. In the context of source-based writing, Zhang et al. (2023) developed and validated a scale to assess L2 source-based writing self-efficacy from a multidimensional perspective. The measure consisted in three dimensions: Self-Regulatory Efficacy (7 items), Discourse Synthesis Self-Efficacy (10 items), and Writing Conventions Self-Efficacy (7 items). Similarly, Zhou et al. (2022) created a multidimensional self-efficacy scale for writing from sources, which included 43 items that evaluated students’ confidence in various writing aspects, such as writing skills, reading comprehension, idea integration, self-regulation strategies, and motivation. In contrast, Bråten et al. (2023) re-conceptualized source-based self-efficacy as a unidimensional construct and developed the Multiple-Source Based Academic Writing Self-Efficacy Scale (MAWSES), an 8-item measure focused uniquely on integrating information from multiple sources in academic writing. At the time of conducting the study, a validated measure for self-efficacy in source-based argumentative writing was lacking.

To measure the students’ self-efficacy in source-based argumentative writing, we reviewed the literature on argumentative and source-based writing and identified five key aspects: (1) evaluating the reliability and strengths of information from various sources; (2) combining sources into coherent arguments and counterarguments; (3) structuring arguments effectively; (4) incorporating evidence from different sources; (5) revising for clarity and grammatical correctness.

Initially, we developed 20 items and phrased them as “I can” statements following guidelines from Bandura (1997) and Pajares et al. (2007). For each statement, respondents indicate their level of agreement or disagreement on a 7-point (1 = never to 7 = always) Likert-type scale. Research indicates that data from 7-point Likert-type items are more accurate than 2, 3, or 4-point scales (Preston and Colman, 2000).

Two expert teachers were invited to examine the items. They reviewed the alignment of the statement with the study’s purpose making sure that the survey statements are focused on a single dimension. After discussion with the expert teachers, one item was removed. The survey was then piloted on 20 students who were not involved in the study. Their comments were used to improve the statements’ readability and avoid any misunderstanding that may arise. Then, we ran exploratory factor analyses on data collected from the pilot test to determine items’ factor loadings and ultimately deleted two items with a value of less than 0.4 (Field, 2009). Factor loadings of the remaining 17 items fit well into the five extracted factors. The reliability of the survey, as measured by Cronbach’s Alpha (0.986), indicates its adequate internal consistency. The final survey is shown in Supplementary Appendix B.

#### 3.3.4 Argumentative source-based writing task

The argumentative writing from sources task is widely used in many academic settings (Cumming et al., 2016). Hence, argumentative writing from sources task was chosen to examine learners’ ability to integrate and synthesize information from multiple source texts. Students were provided with two short passages on global warming and asked students to discuss whether global warming is man-made or natural. They were required to write about 250 words and support their answer with ideas from the two passages. The passages were chosen because they were written on a topic that is familiar to participants and at their reading level. The two passages ranged from 218 to 275 words long, with the same organization pattern and matched readability (Flesch Kincaid Grade level: 11–12; Flesch Reading Ease: 40–60).

#### 3.3.5 The scoring rubric

The evaluation of students’ source-based writing performance was performed using a scoring rubric specifically developed for the study (Supplementary Appendix A). The rubric consisted of four criteria: content, source use, organization, and language use. The same criteria were previously selected by Plakans and Gebril (2013) to analyze students’ performance in integrated writing assignments. These criteria were scored as very good (5), good (4), satisfactory (3), fair (2), poor (1). These levels of accomplishments are commonly used in writing research to assess writing performance (Li and Wang, 2024; Uludag and McDonough, 2022; Wang and Fan, 2021). The rubric was revised by two expert researchers who applied it to a total of 10 student papers from the same course in a previous semester. This revision ensured consistency between raters in measuring students’ performance in writing from sources and resulted in the final rubric version in Supplementary Appendix A. To ensure an accurate evaluation of students’ source-based writing performance, the essays were first rated by the researcher and then by a colleague who was both familiar with and trained in using the scoring rubric. The level of agreement between the first rater and the second rater was assessed using Cohen’s Kappa inter-rater reliability test. The results of the test showed a reliability index of 0.82 which suggests that the scoring was reliable and consistent.

#### 3.3.6 Post-study semi-structured interview

The post-study interview was designed to gather insights from participants regarding their experiences with the scaffolding strategy which involved sequencing assignments and giving feedback on each stage. The interview questions (*n* = 5) focused on various aspects of the intervention including the effectiveness of the scaffolding strategy in guiding students through assignment sequences, the impact of scaffolding on students’ writing performance and self-efficacy in particular, their perceptions of the feedback received at each stage of the scaffolding process, challenges encountered while engaging with the scaffolding strategy, and suggestions for improving the implementation of scaffolding in future assignments.

### 3.4 Procedure

Approval to conduct the study was obtained from the research and ethics committee of [blank] prior to the start of the study. During the first week of the semester, an online self-efficacy survey was administered to assess the participants’ self-efficacy beliefs in source-based argumentative writing. On week 3 and immediately before the introduction of the sequence of assignments, students completed the self-efficacy survey again. The intervention was implemented in the subsequent 12 weeks. Immediately after the intervention, the students were asked to complete the self-efficacy survey as a post-test. After completing the survey, students’ source-based argumentative writing performance was assessed using the argumentative writing task. Students completed the task in class in a timed test condition. Finally, 13 students were invited to participate in a post-study semi-structured interview.

### 3.5 Data analysis

Descriptive statistics were used to describe the sample. Paired-sample *t*-tests were used to test differences from pre-tests 1 and 2 and from pre-test 2 to the post-test. Change scores for pre and post-self-efficacy for individual students were calculated.

Data were analyzed per protocol using IBM SPSS Statistics 23.0. Statistical testing is based on the two-tailed t-test with a significance level set at 0.05. Interview responses were recorded, translated and transcribed. We read students’ responses multiple times to categorize each response into themes that directly address the research question. We looked for recurring ideas and specific examples that highlight students’ perceptions.

## 4 Results

### 4.1 Students’ self-efficacy beliefs

Table 4 presents means and standard deviations on the self-efficacy survey questions for the two pre-tests and one post-test.

**Table 4:** Self-efficacy survey results.

	Pre-test 1		Pre-test 2		Post-test	
	M	SD	M	SD	M	SD
1. I can find the main ideas in different sources.	5.68	1.15	5.54	1.13	6.56	0.76
2. I can understand different opinions presented on a particular topic.	5.08	1.34	5.12	1.14	6.04	1.16
3. I can check if different sources are reliable.	5.34	1.44	5.08	1.51	5.98	1.08
4. I can find weaknesses in the arguments presented in different sources.	4.9	1.53	4.76	1.62	5.08	1.35
5. I can decide if the evidence from different sources is strong.	4.92	1.68	5.06	1.62	5.2	1.50
6. I can put together arguments and counter-arguments from different sources into a new text.	5.16	1.39	4.96	1.41	5.3	1.23
7. I can write new arguments that include different pieces of evidence.	4.94	1.50	4.8	1.41	5.58	1.03
8. I can write counter-arguments to respond to opposite views.	5.14	1.43	5.12	1.47	5.36	1.40
9. I can write a clear thesis statement that shows my final opinion.	5.62	1.24	5.32	1.52	6.22	0.71
10. I can make an outline that organizes my ideas and sources well.	5.3	1.20	4.46	1.30	6.3	0.91
11. I can use evidence from different sources to support my arguments.	5.3	1.30	5.16	1.04	5.56	0.86
12. I can include quotes from different sources to support my arguments.	5.12	1.55	4.92	1.56	5.62	1.29
13. I can paraphrase ideas from different sources using my own words.	5.36	1.44	5	1.51	5.96	0.92
14. I can write a short summary of ideas from different sources.	5.2	1.67	4.84	1.54	6.24	0.94
15. I can correctly cite sources using APA guidelines.	5.54	1.45	5.18	1.59	5.96	1.09
16. I can revise my text to make my arguments and counter-arguments clearer and stronger.	5.06	1.53	4.76	1.57	5.68	1.24
17. I can revise my sentences and paragraphs to eliminate grammar and mechanics errors.	5.26	1.52	4.9	1.64	6.24	1.04
Overall	88.92	18.85	84.68	17.14	98.68	10.74

Three weeks before the intervention, participants reported overall high self-efficacy beliefs (*M* = 88.92, *SD* = 18.85). Participants reported having had a little decrease in their overall self-efficacy beliefs immediately before the intervention, but scores remained positively high. After the interventions, participants reported more positive self-efficacy beliefs in their overall self-efficacy score (*M* = 98.68, *SD* = 10.74). An increase in participants’ self-efficacy beliefs was noted in particular on four statements pertaining to making an outline that organizes ideas and sources well, writing a summary of ideas from different sources, and revising sentences and paragraphs to eliminate grammar and mechanics errors.

To evaluate changes from pre-test 1 to pre-test 2 (before the scaffolded writing intervention), and from pre-test 2 to the post-test (after the scaffolded writing intervention), paired-sample t-tests, were also performed on all 17 dependent measures and the overall self-efficacy score. Results are presented in Table 5.

**Table 5:** Changes in students’ self-efficacy beliefs.

	Pre-test 1 - Pre-test 2				Pre-test 2-Post-test			
	Mean difference	SD	*t*	*sig*.	Mean difference	SD	*t*	Sig.
1. I can find the main ideas in different sources.	0.14	1.37	0.722	0.314	−1.02	1.20	−5.993	<0.001
2. I can understand different opinions presented on a particular topic.	−0.04	1.29	−0.219	0.344	−0.92	1.26	−5.167	<0.001
3. I can check if different sources are reliable.	0.26	1.48	1.241	0.425	−0.9	1.45	−4.4	<0.001
4. I can find weaknesses in the arguments presented in different sources.	0.14	1.77	0.558	0.573	−0.26	0.92	−1.995	0.052
5. I can decide if the evidence from different sources is strong.	−0.14	1.69	−0.586	0.286	−0.1	0.58	−1.219	0.229
6. I can put together arguments and counter-arguments from different sources into a new text.	0.2	1.26	1.121	0.521	−0.3	0.91	−2.333	0.024
7. I can write new arguments that include different pieces of evidence.	0.14	1.34	0.739	0.909	−0.78	1.20	−4.596	<0.001
8. I can write counter-arguments to respond to opposite views.	0.02	1.46	0.097	0.344	−0.18	0.75	−1.703	0.095
9. I can write a clear thesis statement that shows my final opinion.	0.3	1.63	1.3	0.521	−0.9	1.74	−3.656	<0.001
10. I can make an outline that organizes my ideas and sources well.	0.84	1.65	3.582	0.901	−1.84	1.62	−8.027	<0.001
11. I can use evidence from different sources to support my arguments.	0.14	1.22	0.805	0.916	−0.4	0.81	−3.5	0.001
12. I can include quotes from different sources to support my arguments.	0.2	1.91	0.738	0.624	−0.7	1.27	−3.911	<0.001
13. I can paraphrase ideas from different sources using my own words.	0.36	1.58	1.603	0.663	−0.96	1.31	−5.187	<0.001
14. I can write a short summary of ideas from different sources.	0.36	1.68	1.509	1	−1.4	1.46	−6.795	<0.001
15. I can correctly cite sources using APA guidelines.	0.36	1.48	1.718	0.574	−0.78	1.36	−4.057	<0.001
16. I can revise my text to make my arguments and counter-arguments clearer and stronger.	0.3	1.95	1.087	0.301	−0.92	1.29	−5.039	<0.001
17. I can revise my sentences and paragraphs to eliminate grammar and mechanics errors.	0.36	1.74	1.457	1	−1.34	1.35	−7.022	<0.001
Overall	4.24	17.37	1.726	0.631	−14	8.48	−11.673	<0.001

As hypothesized, participants did not show a significant change in their self-efficacy beliefs during the 2 weeks preceding the intervention. Significant changes in self-efficacy beliefs were observed following the intervention on the overall self-efficacy score and on 14 out of the 17 statements. Belief in their abilities to organize, summarize and revise their argumentative increased significantly from pre-test 2 to post-test (i.e., after the scaffolded writing intervention). Differences were statistically significant (*p* = 0.000). Non-significant changes were observed in three statements (I can find weaknesses in the arguments presented in different sources, I can decide if the evidence from different sources is strong, and I can write counter-arguments to respond to opposite views). Overall, as change during the intervention exceeded change before the intervention, this study found evidence that participating in the intervention increased self-efficacy beliefs on source use.

2. Relationship between self-efficacy beliefs and source-based argumentative writing performance.

Table 6 shows the descriptive statistics for measures related to writing performance after the intervention.

**Table 6:** Descriptive statistics for the argumentative source-based writing task.

	Mean	SD	Kurtosis	Skewness	Min.	Max.
Idea development	3.13	0.08	1.03	1.10	1.00	5.00
Organization	4.58	0.88	−0.42	−0.28	2.00	5.00
Source use	4.88	0.99	−1.01	−0.31	2.00	5.00
Language use	1.75	0.85	−1.41	0.53	1.00	3.00
Overall writing performance	14.34	0.82	−1.04	0.34	8	16

The results indicate that students were more successful at integrating evidence from sources and organizing their writing than at using language effectively. Additionally, results revealed that students were less successful at generating ideas and developing their arguments. Language use received lowest scores indicating that students struggled with grammar and mechanics.

One prediction in the study was that the scaffolded intervention may promote students’ self-efficacy and therefore their writing performance. To confirm this prediction, correlational analysis was conducted to see if self-efficacy beliefs were related to students’ writing performance in four major aspects (idea development, organization, source use, and language use). As expected, overall self-efficacy was positively related to source-based argumentative writing performance. Results are displayed in Table 7.

**Table 7:** The correlations between overall self-efficacy beliefs and aspects of source-based writing performance.

	Self-efficacy	Idea development	Organization	Source use	Language use	Writing performance
Self-efficacy	1	0.244	0.356*	0.338*	−0.139	0.304*
*p-*value		0.088	0.011	0.016	0.337	0.032

*Correlations are significant at the 0.05 level.

There is a significantly positive correlation between self-efficacy and source use and organization. Nevertheless, a non-significantly negative correlation was found between self-efficacy and language use. Additionally, the correlation coefficient on the aspect of idea development was moderate but non-significant.

### 4.2 Students’ evaluation of the intervention

It was important to interview the students to gain insights into how the scaffolded writing intervention affected their self-efficacy for source-based argumentative writing. Many students (64%) acknowledged the benefits of the sequence of assignments in enhancing their source-based argumentative writing skills. As one student explained, “starting with a simple one-page topic proposal enabled me to explore topics I find interesting and get a sense of how my essay will be structured.” Another student admitted, “The sequence of assignments helped me preview the literature and see if there are enough sources on the topic, which made me feel more prepared for the final project.” Some students (45%) remarked that providing good reasons in an argumentative essay has never been an easy task before, but with the sequence of assessment they managed to discuss their reasons and read multiple sources which can be used to support their reasons.

Additionally, feedback was highlighted as an essential element in improving their source-based argumentative writing skills. Many students appreciated receiving detailed comments that guided their revisions. One student reported “you asked me to change the sources in the proposal because they were not academic. I was not aware of this. In the annotated bibliography, I used new sources based on your comments.”

Furthermore, the scaffolded writing intervention helped students overcome their frustration with writing a source-based argumentative essay and therefore gain more confidence. As one student noted, “I find writing very challenging but in this course each assessment was connected to next one so I felt less stressed about writing the final essay because I got a chance to fix the issues in the topic proposal and annotated bibliography. Another student was pleased that he received feedback on citations in the first and second assignments: “You told me about my citations in the two assignments so I simply fixed them and copied these into my essay. This saved my time and made me less worried about the correctness of my references.”

Despite the benefits of the scaffolded intervention, a few students reported some challenges with this course. For example, one student found the sequence of related assignments confusing. He asked: “Why do not you just give us one essay project? Why do we need to do so many assignments and submissions?” The student explained that he did not understand that the assessments were connected. Instead of working on the same topic, he ended up choosing different topics for his proposal, annotated bibliography, and argumentative essay. Moreover, the students had some concerns with the time between assignments. As one student explained since I am required to review the first assignment before moving to the next why do not you give us more time to revise our work? Another disadvantage was the lack of teamwork and collaboration. Some students felt that they preferred to work in teams to enhance their writing skills and gain more confidence in their abilities. For example, one student mentioned that team work could enable them to discuss their controversial topic more openly and write a stronger claim that incorporates multiple perspectives. Finally, even though three types of feedback were provided on each assignment, some students were still unsatisfied with the feedback received. One student complained mainly that feedback was not too detailed as the teacher did not highlight all inconsistencies in the assignment.

## 5 Discussion

The aim of the study was to explore the role of self-efficacy-oriented scaffolded intervention in enhancing students’ source-based argumentative writing self-efficacy beliefs and performance, and to investigate the relationship between self-efficacy and source-based writing performance. Additionally, the study sought to obtain students’ opinions on the effectiveness of the intervention, with the goal of informing educators about the potential benefits of scaffolding in improving source-based argumentative writing. Using a double pre-test post-test design, we analyzed the students’ source-based argumentative writing self-efficacy beliefs 3 weeks before the intervention, immediately before the intervention, and immediately after the intervention. This helped assess whether self-efficacy-oriented scaffolded intervention enhanced students’ source-based argumentative writing self-efficacy beliefs. Post-test self-efficacy beliefs were correlated with students’ source-based argumentative writing performance to further understand the role of self-efficacy-based scaffolding. Students’ interview responses provided additional information on the effectiveness of the intervention.

The analyses related to the first research question revealed two significant findings. First, the results demonstrated the important role of designing a self-efficacy-based scaffolding intervention in source-based writing contexts. The overall self-efficacy score improved after the intervention with a medium effect size. These results are likely attributed to the self-efficacy enhancing scaffolds since the participants did not show significant changes in their self-efficacy beliefs before the intervention. Furthermore, these results are not simply attributed to the practice effect because we used alternative formats of assignments and gave a minimum of three-week intervals between pre-test and post-test.

Second, further evidence demonstrated that the intervention had a positive and significant impact in particular on students’ self-efficacy beliefs of their abilities to organize ideas, summarize different sources, and revise their essays. This significant improvement could imply that the scaffolding activities prompted students to focus more on their source use and revision skills. It is likely that the structured sequence of assignments and the deliberate focus on writing annotations, outlining, and revising helped students pay more attention to their abilities in those areas which resulted in an increase in their self-efficacy beliefs. However, there were no significant changes in their beliefs about skills related to deciding if the evidence from different sources is strong, finding weaknesses in the arguments presented in different sources, and writing a counterargument. These skills may be a more complex and challenging requiring more extensive practice, instruction, and feedback to develop. Students’ self-efficacy beliefs in these skills may have been more resistant to change, as they still lacked a full understanding of the nuances and strategies involved in constructing effective arguments. It is also possible that students at the outset overestimated their abilities in writing a counter-argument and selecting the appropriate evidence and hence they did not perceive a need to develop their skills further.

Our results partly align with findings from previous studies in showing that self-efficacy-based scaffolding gave students the support they needed to accomplish complex assignments such as a source-based argumentative essay (Cui et al., 2021; Ha et al., 2021; Huang et al., 2020; Koskinen et al., 2023; Li et al., 2023; Mandouit and Hattie, 2023 Van Blankenstein et al., 2019; Zhou et al., 2022). This support led to an overall increase in their self-efficacy beliefs and specifically enhanced their skills in using sources and revising their work. Thus, our results confirm the idea that self-efficacy beliefs are context-dependent. Various factors, such as the educational background, the type of instructional support, the genre of writing, and the level of student engagement with feedback may affect self-efficacy (Lundin et al., 2023; Mitchell and McMillan, 2018).

The second research question attempted to explore an important correlate of self-efficacy beliefs. The results revealed a positive relationship between self-efficacy beliefs and source-based argumentative writing performance. Correlational analysis was conducted using post-test self-efficacy overall score and students’ writing performance scores on four major aspects (idea development, organization, source use, and language use). Results supported the idea that self-efficacy-based scaffolding may enhance students’ writing performance. Students who ended the intervention with higher self-efficacy beliefs had high scores on organization and source use. This suggests that the intervention effectively supported students in enhancing their ability to structure their ideas, summarize information, paraphrase effectively, and produce well-integrated essays, potentially leading to improved performance in these specific areas. These results echo the extensive literature on the positive relationship between writing self-efficacy and writing performance (e.g., Bruning and Kauffman, 2016; Camacho et al., 2021; Klassen, 2002; Pajares, 2003; Pajares et al., 2007; Zhou et al., 2022). While students showed improvement in their self-efficacy beliefs about revising sentences and paragraphs to address grammar and mechanics errors, the correlation between their overall self-efficacy score and language use was negative and not statistically significant. This result may be attributed to the timed writing test condition which may have influenced their performance in the language area.

Students’ interview results revealed that the scaffolded writing intervention had a positive impact on their self-efficacy and performance in source-based argumentative writing. In terms of writing performance, many students reported that the sequence of assignments helped them enhance their skills, particularly in choosing a topic, previewing literature, and using multiple sources to support their arguments. Feedback was highlighted as essential in improving their writing skills, with many students appreciating detailed comments that guided their revisions.

In terms of self-efficacy, students reported that the scaffolded intervention helped them overcome their frustration with writing a source-based argumentative essay and gain more confidence. Students appreciated the opportunity to fix issues in earlier assignments, which reduced their stress about writing the final essay. However, some students reported some challenges including confusion with the sequence of assignments and the time between submissions. Additionally, some students felt that teamwork and collaboration should have been encouraged to enhance their writing skills and confidence. These findings are also in line with the literature on students’ satisfaction with interventions designed to enhance writing self-efficacy (Britt and Aglinskas, 2002; Cui et al., 2021; Doolan and Fitzsimmons-Doolan, 2023; Lee and Evans, 2019; Li et al., 2023; Luna et al., 2023; Mateos et al., 2008).

## 6 Implications, limitations, and future research

Several theoretical, practical and methodological implications can be drawn from the findings in this study. On a theoretical level, Bandura’s (1997) theory of self-efficacy emphasizes that individuals’ beliefs in their capabilities significantly influence their motivation, performance, and persistence. According to Bandura (1997), self-efficacy beliefs are malleable and can be shaped by four primary sources (mastery experiences, vicarious experiences, social persuasion, emotional states). The findings from this study contributed to an understanding of the impact of self-efficacy-based scaffolding strategies in the context of learning how write a source-based argumentative essay in an L2 classroom environment.

On a practical level, our findings suggest that teachers should integrate all four sources of self-efficacy into their writing assignments. To help students manage stress and anxiety, teachers could provide strategies and support mechanisms, such as mindfulness exercises or stress-reduction strategies. Additionally, teachers should offer vicarious experiences by modeling effective writing strategies through exemplars and demonstrations. Providing opportunities for practice after each modeled example will enhance students’ mastery experiences, allowing them to internalize and apply new skills. Finally, offering customized feedback will enable students to track their progress and receive encouragement, thereby reinforcing their self-efficacy and fostering ongoing improvement.

Methodologically, the study offers a novel and practical paradigm for experimental research on self-efficacy, employing a double pre-test post-test design without a control group. The study’s design can serve as a model for other researchers aiming to evaluate the effectiveness of interventions on self-efficacy. It demonstrates a practical approach to capturing the effect of self-efficacy based scaffolding interventions, making it a valuable tool for experimental research in educational and psychological contexts.

One limitation of this study is its focus on three main scaffolding strategies (teacher feedback, models, and sequencing). While this approach effectively integrated Bandura’s four sources of self-efficacy: vicarious experience, mastery experience, social persuasion, and emotional states, it did not examine other instructional strategies that could also align with these four sources of self-efficacy, such as gamified learning, self-assessment, and stress and anxiety management strategies. Future research could further explore the effectiveness of these additional strategies in enhancing self-efficacy.

Another limitation in this study is the lack of consideration of various contextual factors that can influence the effectiveness of scaffolding interventions. While the results are promising, the study did not take into account all factors that can influence the success of scaffolding interventions such as the cultural, geographical, and personality characteristics of the participants. For example, individual differences in personality traits like introversion/extraversion may affect how students respond to scaffolding and their overall engagement in the learning process (Banaruee et al., 2023). Similarly, cultural norms and values may shape students’ learning preferences, perceptions of self-efficacy, and responses to scaffolding strategies (Banaruee et al., 2023). Considering these factors in future research can help provide a more comprehensive picture on the impact of scaffolding on enhancing students’ self-efficacy beliefs and writing performance.

Finally, the study explored the impact of scaffolding on self-efficacy in one genre. To gain a better understanding of self-efficacy, future research should investigate how self-efficacy beliefs might vary across different genres. For example, examining how self-efficacy changes in narrative versus argumentative writing could reveal insights into the domain-specific nature of self-efficacy beliefs. This would help clarify how self-efficacy influences performance in various genres and how genre-specific factors might interact with self-efficacy-based interventions.

## Data Availability

The raw data supporting the conclusions of this article will be made available by the authors, without undue reservation.
