# Supplementary figures and images for: A scaffolding intervention to improve self-efficacy in source-based argumentative writing
Source: Front Psychol. 2024 Nov 8;15:1454104. doi: 10.3389/fpsyg.2024.1454104 (PMC11581951; doi:10.3389/fpsyg.2024.1454104)

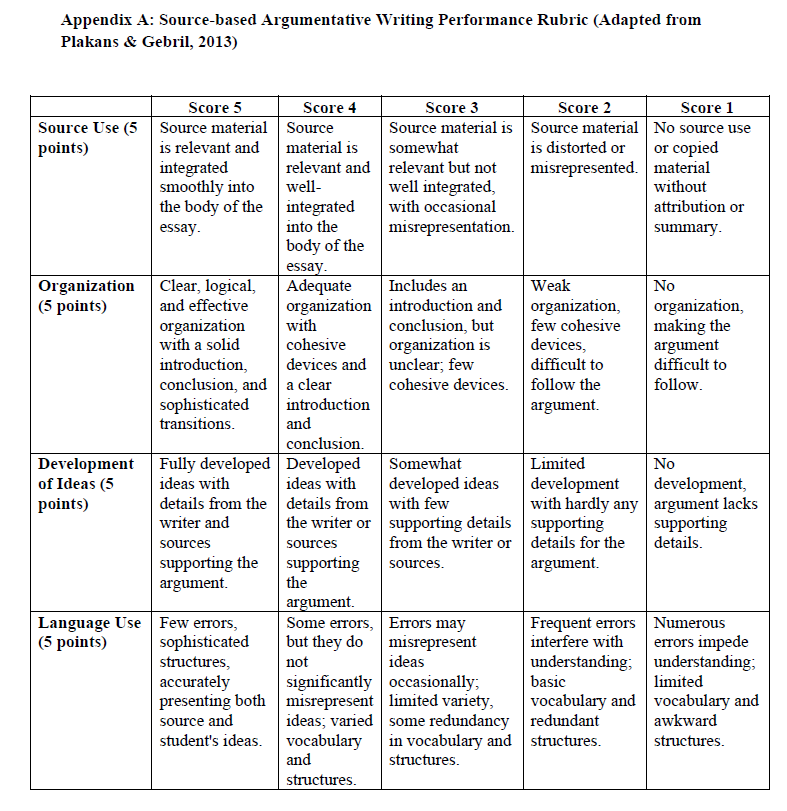

Supplement: Supplementary file 1 [file Image_1.png]

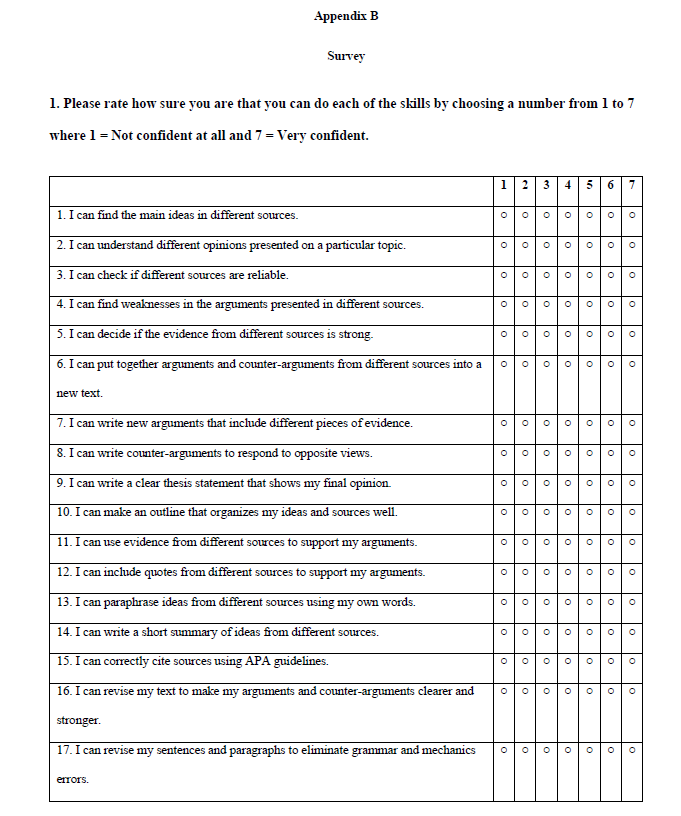

Supplement: Supplementary file 2 [file Image_2.png]
